# Revealing metabolite biomarkers for acupuncture treatment by linear programming based feature selection

**DOI:** 10.1186/1752-0509-6-S1-S15

**Published:** 2012-07-16

**Authors:** Yong Wang, Qiao-Feng Wu, Chen Chen, Ling-Yun Wu, Xian-Zhong Yan, Shu-Guang Yu, Xiang-Sun Zhang, Fan-Rong Liang

**Affiliations:** 1 Academy of Mathematics and Systems Science, Chinese Academy of Sciences, Beijing 100190, China; 2 Acupuncture and Moxibustion College, Chengdu University of Traditional Chinese Medicine, Chengdu 610075, China; 3 National Center for Biomedical Analysis, Beijing 100850, China; 4 National Center for Mathematics and Interdisciplinary Sciences, Chinese Academy of Sciences, Beijing 100190, China

## Abstract

**Background:**

Acupuncture has been practiced in China for thousands of years as part of the Traditional Chinese Medicine (TCM) and has gradually accepted in western countries as an alternative or complementary treatment. However, the underlying mechanism of acupuncture, especially whether there exists any difference between varies acupoints, remains largely unknown, which hinders its widespread use.

**Results:**

In this study, we develop a novel Linear Programming based Feature Selection method (LPFS) to understand the mechanism of acupuncture effect, at molecular level, by revealing the metabolite biomarkers for acupuncture treatment. Specifically, we generate and investigate the high-throughput metabolic profiles of acupuncture treatment at several acupoints in human. To select the subsets of metabolites that best characterize the acupuncture effect for each meridian point, an optimization model is proposed to identify biomarkers from high-dimensional metabolic data from case and control samples. Importantly, we use nearest centroid as the prototype to simultaneously minimize the number of selected features and the leave-one-out cross validation error of classifier. We compared the performance of LPFS to several state-of-the-art methods, such as SVM recursive feature elimination (SVM-RFE) and sparse multinomial logistic regression approach (SMLR). We find that our LPFS method tends to reveal a small set of metabolites with small standard deviation and large shifts, which exactly serves our requirement for good biomarker. Biologically, several metabolite biomarkers for acupuncture treatment are revealed and serve as the candidates for further mechanism investigation. Also biomakers derived from five meridian points, Zusanli (ST36), Liangmen (ST21), Juliao (ST3), Yanglingquan (GB34), and Weizhong (BL40), are compared for their similarity and difference, which provide evidence for the specificity of acupoints.

**Conclusions:**

Our result demonstrates that metabolic profiling might be a promising method to investigate the molecular mechanism of acupuncture. Comparing with other existing methods, LPFS shows better performance to select a small set of key molecules. In addition, LPFS is a general methodology and can be applied to other high-dimensional data analysis, for example cancer genomics.

## Background

Acupuncture, an important therapeutic method in Traditional Chinese Medicine (TCM), has been used to treat various diseases for thousand years in China. Recently it has been gradually accepted in western countries as an alternative or complementary treatment. However, how the acupuncture works remains an open question though acupuncture exists as one of the oldest continuous systems of medicine dating back 4,000 years. Extensive studies have been conducted on the mechanism of acupuncture to explain the effects of acupuncture on various systems and symptoms [1-3]. Compared to the relatively widespread use of acupuncture, systems biology is a new term to describe the recent trends in biology research. It emphasizes the high-throughput measurement of biological systems and focuses on the complex interactions in biological systems [4,5]. We highly expect that systems biology, a biology-based inter-disciplinary study field, will provide tremendous opportunities for revealing acupuncture mechanism at the molecular level.

In this paper, we use systems biology method to study the acupuncture treatment effect by identifying a subset of important molecules from high-throughput metabolic data. Specifically, we separate the acupuncture from moxibustion and only study the effect of acupuncture on normal people by investigating the difference between acupuncture at particular acupoint and without acupuncture. Towards this aim, we utilize ^1^H nuclear magnetic resonance (^1^H NMR) to investigate the effects of acupuncture at several meridian points on plasma metabolites. Then metabolite profiles (vectors) are generated from a collection of case samples(with acupuncture at meridian point) and control samples (without acupuncture). These high-dimensional profile data is very similar to SNP (sequence data), gene expression (transcriptome), mass spectrum (proteome), and small molecules (metabolome) data in different levels. Then the straightforward task is to identify differentially expressed molecules and further classify and predict the diagonostic category of a sample, based on its metabolite profile [6].

Generally speaking, there are two difficulties in analyzing these high-dimensional profile data. First, a large number of features (metabolites in our case) are available to predict classes for a relatively small number of samples. The presence of a significant number of irrelevant features that are unrelated to the case status makes such analysis prone to the curse of dimensionality. Second, predictive accuracy is not the only goal and further biological validation and mechanism understanding call for explanatory power other than black box predictive results. Thus it is especially important to know which molecules largely contribute towards the classification. Ideally we can improve the generalization performance of classifier by identifying only the molecules that are significantly contribute to the classifier. This effect is attributable to the overcoming of the curse of dimensionality. For example, if it is possible to identify a small set of metabolites that is indeed capable of providing complete discriminatory information, inexpensive diagnostic assays for only a few metabolites might be developed and be widely deployed in clinical settings. Knowledge of a small set of diagnostically relevant metabolites may provide important insights into the mechanisms responsible for acupuncture treatment itself. Those molecules are usually termed as biomarkers. The procedure to reveal them is referred as feature selection, biomarker identification, or feature ranking.

Feature selection is known to be NP-hard [7] and the search becomes quickly computationally intractable. Suppose we treat the feature selection task in a brute force way. Given *n *features, we need to select *m *features which can get the best classification accuracy (*m *<<*n*) regarding to a predefined cost function. Usually in classification or prediction problem, the cost function is selected as the accuracy of the prediction. The exhaustive search method goes through all the possible combinations, with the computation complexity *O*(*n*^*m*^). Thus, this method is not practical for realistic applications.

Existing feature selection strategies can be roughly categorized into three types [8]. Considering the partial ordering properties of the subset space, we can either start with an empty set and successively add features, or start with the set of all features and successively filter them. The former type is referred to as forward selection while the latter is referred to as backward elimination. The third type is the combination of the two approaches. However, all the above methods relies on the greedy strategy. As an example of forward feature selection, we might first look for the single most discriminative feature using any classifier. Then we could search the single additional feature that gives the best class discrimination when considered along with the first feature. Keeping augmenting the feature set iteratively in this greedy fashion, we stop until cross-validation error estimates are minimized. As a result, the global optimal solution usually cannot be guaranteed.

In this paper, we proposed a novel linear programming (LP) model to address this important problem. Feature selection problem is cast into an optimization problem with two objectives, one is to minimize the number of chosen features and the other is to maximize the predictive accuracy based on the centroid classification framework. In other words, our feature selection method simultaneously improves classification accuracy and selects features. Comparing with several state-of-the-art feature selection methods, our Linear Programming based Feature Selection (LPFS) method can select a small set of features by applying strong regularization while keeping high accuracy. We then apply our method to analyze the metabolite profile data generated for acupuncture treatment. We identify important molecules (biomarkers) related to the acupuncture treatment for several meridian points. Further characterization of the biomarkers and the common and difference among several meridian points provide biological insights for acupuncture mechanisms at molecular level. Preliminary results in this paper were presented in our conference paper [9]. In this extended paper, we further provide the detailed derivation of the method and comparison with existing works. In addition, we performed more analysis and descriptions for the biological insights of the acupuncture biomarkers.

## Method

### Analytic workflow

**Figure 1:**
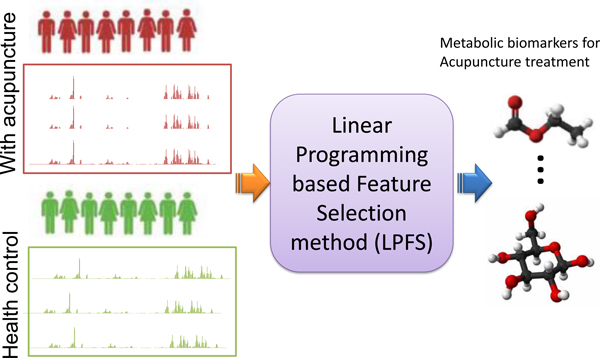
**Our analytic workflow to identify biomarkers for acupuncture treatment effect**. Metabolite profiles are originally generated by ^1^H NMR from control samples and the samples with acupuncture treatment. Then we develop a linear programming based feature selection method to compare the two groups of metabolite profiles. Finally a small set of metabolites are selected as biomarkers for acupuncture effect.

In this paper, the acupuncture treatment effect is investigated in the framework of systems biology. The basic analytic workflow is shown in Figure 1. As the first step, metabolite profiles are originally generated by ^1^H NMR from control samples and the samples with acupuncture treatment in meridian points. Then we develop a linear programming based feature selection method to compare the two groups of metabolite profiles. Finally, a small set of metabolites are selected as biomarkers for acupuncture treatment effect.

### Overview of the linear programming based feature selection

**Figure 2:**
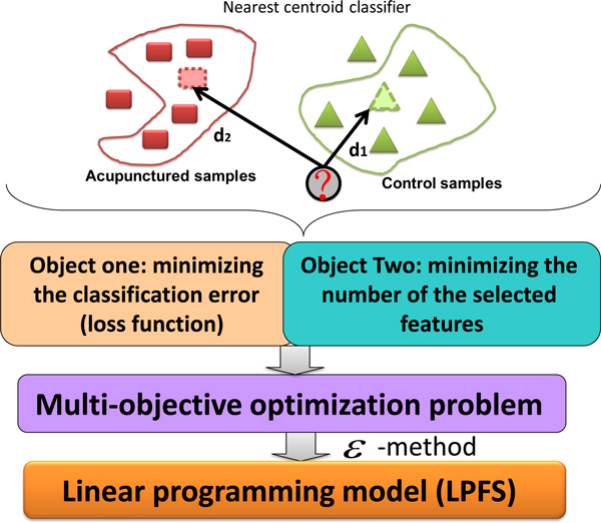
**The schematic illustration of LPFS**. Under the nearest centroid framework, LPFS requires to minimize the classification error and the number of selected features, which leads to a multi-objective programming. To ensure the computational efficiency, a linear programming is solved to identify biomarkers.

To investigate the high-dimensional data for acupuncture treatment effect, we develop a novel method, LPFS, to select a small set of metabolites to characterize acupuncture treatment effect. The schematic illustration of LPFS is shown in Figure 2. LPFS performs feature selection based on the nearest centroid classifier. On one hand, we want to attain the best classification accuracy by minimizing the loss function. On the other hand, feature selection algorithms should be robust to noise and outliers in the data by applying strong regularization. Here we use the parsimony principle (also known as Occam's razor) by minimizing the number of selected features. Then the feature selection problem is formulated as a multi-objective programming. The next step is to convert this multi-objective programming into a single-objective linear programming by applying the *ε *method. After solving the linear programming model in an efficient way, the optimal features can be selected.

#### Centroid classification prototype

A fast and simple algorithm for classification is the centroid method [6,10]. This algorithm assumes that the target classes correspond to individual (single) clusters and uses the cluster means (or centroids) to determine the class of a new sample point (see Figure 2). A prototype pattern for class *C*_*j *_is defined as the arithmetic mean:

(1) $$\mu_{C_{j}}=\frac{1}{\left|C_{j}\right|}\underset{s_{i}\in C_{j}}{\sum }x_{i}$$

where ***s***_*i *_is the *i*-th training sample labeled as class *C*_*j*_. Recall that the training sample is a metabolite spectra represented as a multi-dimensional vector (denoted in bold). In a similar fashion, we can obtain a prototypical vector for all the other classes. During classification, the class label of an unknown sample ***s ***is determined as:

(2) $$C\left(s\right)=\text{arg}\underset{C_{j}}{\text{min}}dis\left({\mu_{C}}_{{}_{j}},s\right)$$

where *dis*(***x***, ***y***) is a distance function or:

(3) $$C\left(s\right)=\text{arg}\underset{C_{j}}{\text{max}}sim\left({\mu_{C}}_{{}_{j}},s\right)$$

where *sim*(***x***, ***y***) is a similarity metric. This simple classifier will form the basis of our LPFS method. The advantages is that it works with any number of features. And its run-time complexity is proportional to the number of features and the complexity of the distance or similarity metric used. According to the experiments in [11], we select *L*_1 _distance metric, which is robust to outliers and most appropriate for the centroid classification algorithm. It is defined by:

(4) $$L_{1}\left(s,\mu \right)={∥s-\mu ∥}_{1}$$

with ║*y*║_1 _= ∑_*i *_|*y*(*i*)|, and *y*(*i*) being the i-th element of vector ***y***. The value *L*_1_(***s***, ***μ***) has a linear cost in the number of features. In this study, data sets contain two classes and hence the number of calls to the distance metric is also two. Therefore, the centroid classifier, at run-time, is linear in the number of features. During training, two prototypes are computed and the cost of computing each prototype is *O*(*mN*), where *N *is the number of features and *m *is the number of training samples which belong to a given class. Note that *m *only varies between data sets and not during training or feature selection processes. Thus, we can view *m *as a constant and the centroid classifier has *O*(*N*) cost in the training phase.

#### Multi-objective optimization model

Suppose we have two groups in the training dataset, the case group and the control group as the gold-standard data to classify new samples. We denote them set *T *and *F *respectively. Supposing |*T| *= *m*_1_, |*F| *= *m*_2_, and the computed centroids are ***μ***_*T *_and ***μ***_*F *_respectively. A simple classification scheme is as follows. Given a new sample ***s***, we want to decide which group it belongs to. The *L*_1 _discrepancy between the sample ***s ***and the groups *T *and *F *can be calculated as ║***s ***- ***μ***_*T*_║_1 _and ║***s ***- ***μ***_*T*_║_1_. Thus a simple rule is

(5) $$s\in T\;\text{if}\;{∥s-\mu_{T}∥}_{1}<{∥s-\mu_{F}∥}_{1}$$

(6) $$s\in F\;\text{if}\;{∥s-\mu_{T}∥}_{1}>{∥s-\mu_{F}∥}_{1}$$

Let the feature number be *n*. We introduce the variables for feature selection as ***x ***= (*x*_1_, *x*_2_, ⋯, *x*_*n*_), where *x*_*i *_= 0,1. When *x*_*i *_= 1, it means feature *i *is selected in the biomarker set. Otherwise it is not selected.

Suppose the test dataset is *U*. And it is composed by the case group *U*_*T *_and control group *U*_*F*_. *U *= *U*_*T *_∪ *U*_*F*_, and |*U*_*T*_| = *l*_1_, |*U*_*F*_| = *l*_2_. Given a case sample ***s***_***l ***_= (*s*_*l*1_, *s*_*l*2_, ⋯, *s*_*ln*_), *l *∈ {1, 2 ⋯, *l*_1_}, if it is classified correctly, we should have

(7) $$\overset{n}{\underset{i=1}{\sum }}\left|s_{li}-\overset{m_{1}}{\underset{j=1}{\sum }}t_{ji}/m_{1}\right|x_{i}<\overset{n}{\underset{i=1}{\sum }}\left|s_{li}-\overset{m_{2}}{\underset{j=1}{\sum }}f_{ji}/m_{2}\right|x_{i}$$

Where ***t***_*k *_= (*t*_*k*1_, *t*_*k*2_, ⋯, *t*_*kn*_) ∈ *T*, *k *= 1, 2, ⋯, *m*_1 _and ***f***_*k *_= (*f*_*k*1_, *f*_*k*2_, ⋯, *f*_*kn*_) ∈ *F, k *= 1, 2, ⋯, *m*_2_.

Similarly given a control sample ***s***_***l ***_= (*s*_*l*1_, *s*_*l*2_, ⋯, *s*_*ln*_), *l *∈ {*l*_1 _+ 1, *l*_1 _+ 2 ⋯, *l*_1 _+ *l*_2_}, if it is classified correctly, we should have

(8) $$\overset{n}{\underset{i=1}{\sum }}\left|s_{li}-\overset{m_{1}}{\underset{j=1}{\sum }}t_{ji}/m_{1}\right|x_{i}>\overset{n}{\underset{i=1}{\sum }}\left|s_{li}-\overset{m_{2}}{\underset{j=1}{\sum }}f_{ji}/m_{2}\right|x_{i}$$

With the above constraints for variable ***x ***= (*x*_1_, *x*_2_, ⋯, *x*_*n*_), the objective function is to choose as few as features, i. e.,

(9) $$\underset{x_{1},x_{2},\cdots \;,x_{n}}{\text{min}}\overset{n}{\underset{i=1}{\sum }}x_{i}$$

Thus the feature selection problem is formulated as an integer linear programming problem in Equation (10).

(10) $$\begin{array}{c} \;\underset{x_{1},x_{2},\cdots \;,x_{n}}{\text{min}}\;\;\;\;\;\;\;\;\;\;\;\sum_{i=1}^{n}x_{i}\\ s.t.\;\;\;\sum_{i=1}^{n}\left|p_{li}-\sum_{j=1}^{m_{1}}t_{ji}/m_{1}\right|x_{i}<\sum_{i=1}^{n}\left|p_{li}-\sum_{j=1}^{m_{2}}f_{ji}/m_{2}\right|x_{i},\\ \;\;\;\;\;\;p_{l}=\left(p_{l1},s_{l2},\cdots \;,p_{ln}\right)\in U_{T},l\in \left\{1,2,\cdots \;,l_{1}\right\},\\ \;\;\;\;\sum_{i=1}^{n}\left|p_{li}-\sum_{j=1}^{m_{1}}t_{ji}/m_{1}\right|x_{i}>\sum_{i=1}^{n}\left|p_{li}-\sum_{j=1}^{m_{2}}f_{ji}/m_{2}\right|x_{i}\\ \;\;\;\;\;\;p_{l}=\left(p_{l1},s_{l2},\cdots \;,p_{ln}\right)\in U_{F},l\in \left\{1,2,\cdots \;,l_{2}\right\},\\ \;\;\;\;\;\;\;\;\;\;x_{i}=0,1,\;i\in \left\{1,2,\cdots \;,n\right\} \end{array}$$

When we consider the noise in the measured data, not all the test samples can be classified exactly. We introduce the tolerable error ***y ***= {*y*_1_, *y*_2_, ..., *y_l_*_1_*_+l_*_2_} for every sample in *U*_*T *_∪ *U*_*F*_. And *y*_*i *_≥ 0, *i* ε {1,2, ..., *l*_1_ + *l*_2_}. When *y*_*i *_is not equal to zero, it means sample *i *is wrongly classified. Otherwise this sample should be correctly classified.

Given a case sample ***s***_***l ***_= (*s*_*l*1_, *s*_*l*2_, ⋯, *s*_*ln*_), *l *∈ {1, 2 ⋯, *l*_1_}, we should have the following constraint considering the tolerable error

(11) $$\overset{n}{\underset{i=1}{\sum }}\left|s_{li}-\overset{m_{1}}{\underset{j=1}{\sum }}t_{ji}/m_{1}\right|x_{i}-y_{l}<\overset{n}{\underset{i=1}{\sum }}\left|s_{li}-\overset{m_{2}}{\underset{j=1}{\sum }}f_{ji}/m_{2}\right|x_{i}$$

Similarly given a control sample ***s***_***l ***_= (*s*_*l*1_, *s*_*l*2_, ⋯, *s*_*ln*_), *l *∈ {*l*_1 _+ 1, *l*_1 _+ 2 ⋯, *l*_1 _+ *l*_2_}, we should have the following constraint considering the tolerable error

(12) $$\overset{n}{\underset{i=1}{\sum }}\left|s_{li}-\overset{m_{1}}{\underset{j=1}{\sum }}t_{ji}/m_{1}\right|x_{i}+y_{l}>\overset{n}{\underset{i=1}{\sum }}\left|s_{li}-\overset{m_{2}}{\underset{j=1}{\sum }}f_{ji}/m_{2}\right|x_{i}$$

Thus the objective function composes two parts, i. e., we want to choose as few as features ${\text{min}}_{x_{1},x_{2},\cdots \;,x_{n}}\overset{n}{\underset{i=1}{\sum }}x_{i}$ and at the same time we want to reduce the classification error (loss function) $\underset{y_{1},y_{2},\cdots \;,y_{l_{1}+l_{2}}}{\text{min}}\sum_{i=1}^{l_{1}+l_{2}}y_{i}$. In general, there is a trade-off relationship between the classification error and the number of features. Hence, the feature selection problem can be formulated as a multi-objective optimization problem with discrete variables ***x ***= (*x*_1_, *x*_2_, ⋯, *x*_*n*_) and continuous variables $y=\left(y_{1},y_{2},\cdots \;,y_{l_{1}+l_{2}}\right)$ as shown in Equation (13).

(13) $$\begin{array}{c} \text{vector - minimiz}{\text{e}}_{\left(x,y\right)}\;\;\;\;\;\;\;\left\{\sum_{i=1}^{n}x_{i},\sum_{i=1}^{l_{1}+l_{2}}y_{i}\right\},\\ \;\;\;\;\;\;\text{subject}\;\text{to}\;\;\left(11\right)\left(12\right)\;\text{with}\;x_{i}\in \left\{0,1\right\},i\in \left\{1,2,\cdots \;,n\right\},\\ \;\;\;\;\;\;\;\;\;\;\;\;\;\;y_{i}\geq 0,i\in \left\{1,2,\cdots \;,l_{1}+l_{2}\right\} \end{array}$$

The first term of objective function in Equation (13) is to minimize the number of chosen features, and the second one is to minimize the total classification error.

#### Mixed integer linear programming

The optimal solutions of the two-objective optimization problem consist of a Pareto set, which can be solved by transforming the two objectives of (13) into a single objective. One typical technique is the *ϵ*-method, which alternates a positive scalar parameter *λ *to obtain the Pareto set, with the formulation in Equation (14).

(14) $$\begin{array}{c} \underset{x,y}{\text{min}}\;\;\;\;\;\;\;\;\sum_{i=1}^{n}x_{i}+\lambda \sum_{i=1}^{l_{1}+l_{2}}y_{i}\\ s.t.\;\sum_{i=1}^{n}\left|p_{li}-\sum_{j=1}^{m_{1}}t_{ji}/m_{1}\right|x_{i}-y_{l}<\sum_{i=1}^{n}\left|p_{li}-\sum_{j=1}^{m_{2}}f_{ji}/m_{2}\right|x_{i}\\ \;\;\;\;\;\;p_{l}=\left(p_{l1},s_{l2},\cdots \;,p_{ln}\right)\in U_{T},l\in \left\{1,2,\cdots \;,l_{1}\right\},\\ \;\;\sum_{i=1}^{n}\left|p_{li}-\sum_{j=1}^{m_{1}}t_{ji}/m_{1}\right|x_{i}+y_{l}>\sum_{i=1}^{n}\left|p_{li}-\sum_{j=1}^{m_{2}}f_{ji}/m_{2}\right|x_{i}\\ \;\;\;\;\;\;p_{l}=\left(p_{l1},s_{l2},\cdots \;,p_{ln}\right)\in U_{F},l\in \left\{1,2,\cdots \;,l_{2}\right\},\\ \;\;x_{i}=0,1,\;i\in \left\{1,2,\cdots \;,n\right\},y_{j}\geq 0,\;j\in \left\{1,2,\cdots \;,l_{1}+l_{2}\right\} \end{array}$$

(14) is a mixed integer programming (ILP). The objective function in (14) is $\sum_{i=1}^{n}x_{i}+\lambda \sum_{i=1}^{l_{1}+l_{2}}y_{i}$. Theoretically, we can obtain all optimal solutions belonging to the Pareto set by changing the parameter *λ *for the single-objective optimization problem (14). Clearly, *λ *transforms the number of chosen features into equivalent classification error in (14), and controls the balance between them.

By solving the proposed mixed integer linear programming model (14), we can get solutions for the feature selection variables *x*_*i*_, *i *∈ {1, 2, *· · *·, *n*}, and classification error variables *y*_*j*_, *j *∈ {1, 2, ⋯, *l*_1 _+ *l *_2_}. Checking if *x*_*i *_is equal to 1, we can know if the corresponding feature should be selected in the classifier. Meanwhile checking the values of all the *y*_*j*_, we can estimate the classification accuracy. For example, suppose the number of all *j *such that *y*_*j *_= 0 is *N*_1 _and the number of all *j *such that *y*_*j *_> 0 is *N*_2_. We can simply calculate the classification accuracy by *N*_1_/*l*_1_+*l*_2 _and *N*_2_/*l*_1_+*l*_2_.

#### Leave-one-out cross validation

The above model (14) is based on the general idea of cross validation, thus it depends on the choice of *T *and *F*. We noticed that there are different ways to do cross validation in feature selection [12]. One way is that the feature selection is done with all the samples and the cross-validation is only done for the classification procedure. This kind of cross validation may severely bias the evaluation in favor of the studied method due to "information leak" in the feature selection step. Another way is to include the feature selection procedure in the cross validation, i.e., to leave the test sample(s) out from the training set before undergoing any feature selection. Our feature selection model allows the freedom to choose the suitable cross validation procedure according to the practical need. If more information is preferred to select biomarkers due to the scarcity of samples, we can use all the samples to estimate the cross validation error in the following resubstitution validation in Equation (15).

(15) $$\begin{array}{c} \underset{x,y}{\text{min}}\;\;\;\;\;\;\;\;\sum_{i=1}^{n}x_{i}+\lambda \sum_{i=1}^{l_{1}+l_{2}}y_{i}\\ s.t.\;\sum_{i=1}^{n}\left|p_{li}-\sum_{j=1}^{m_{1}}t_{ji}/m_{1}\right|x_{i}-y_{l}<\sum_{i=1}^{n}\left|p_{li}-\sum_{j=1}^{m_{2}}f_{ji}/m_{2}\right|x_{i}\\ \;\;\;\;\;\;p_{l}=\left(p_{l1},s_{l2},\cdots \;,p_{ln}\right)\in T,l\in \left\{1,2,\cdots \;,m_{1}\right\},\\ \;\;\sum_{i=1}^{n}\left|p_{li}-\sum_{j=1}^{m_{1}}t_{ji}/m_{1}\right|x_{i}+y_{l}>\sum_{i=1}^{n}\left|p_{li}-\sum_{j=1}^{m_{2}}f_{ji}/m_{2}\right|x_{i}\\ \;\;\;\;\;\;p_{l}=\left(p_{l1},s_{l2},\cdots \;,p_{ln}\right)\in F,l\in \left\{1,2,\cdots \;,l_{2}\right\},\\ \;\;x_{i}=0,1,\;i\in \left\{1,2,\cdots \;,n\right\},y_{j}\geq 0,\;j\in \left\{1,2,\cdots \;,l_{1}+l_{2}\right\} \end{array}$$

Resubstitution error rate indicates only how good are our biomarkers on the training data. However, this model has "information leak" and will underestimate the classification error. In our implement, we choose the model for leave-one-out cross validation in Equation (16).

(16) $$\begin{array}{c} \underset{x,y}{\text{min}}\;\;\;\;\;\;\;\;\;\;\;\;\;\;\;\;\sum_{i=1}^{n}x_{i}+\lambda \sum_{i=1}^{l_{1}+l_{2}}y_{i}\\ s.t.\;\;\;\;\;\;\;\;\sum_{i=1}^{n}\left|p_{li}-\sum_{j=1}^{m_{1}-1}t_{ji}/\left(m_{1}-1\right)\right|x_{i}-y_{l}<\sum_{i=1}^{n}\left|p_{li}-\sum_{j=1}^{m_{2}}f_{ji}/m_{2}\right|x_{i}\\ \;\;p_{l}=\left(p_{l1},s_{l2},\cdots \;,p_{ln}\right)\in T,l\in \left\{1,2,\cdots \;,l_{1}\right\},t_{k}=\left(t_{k1},t_{k2},\cdots \;,t_{kn}\right)\in T\backslash \left\{p_{l}\right\},k\in \left\{1,2,\cdots \;,m_{1}\right\}\backslash \left\{l\right\}\\ \;\;\;\;\;\;\;\;\;\sum_{i=1}^{n}\left|p_{li}-\sum_{j=1}^{m_{1}}t_{ji}/m_{1}\right|x_{i}+y_{l}>\sum_{i=1}^{n}\left|p_{li}-\sum_{j=1}^{m_{2}-1}f_{ji}/\left(m_{2}-1\right)\right|x_{i}\\ \;\;p_{l}=\left(p_{l1},s_{l2},\cdots \;,p_{ln}\right)\in F,l\in \left\{1,2,\cdots \;,l_{2}\right\},f_{k}=\left(f_{k1},f_{k2},\cdots \;,f_{kn}\right)\in F\backslash \left\{p_{l}\right\},k\in \left\{1,2,\cdots \;,m_{2}\right\}\backslash \left\{l\right\}\\ \;\;\;\;\;\;\;\;\;\;\;x_{i}=0,1,\;i\in \left\{1,2,\cdots \;,n\right\},y_{j}\geq 0,\;j\in \left\{1,2,\cdots \;,l_{1}+l_{2}\right\} \end{array}$$

We adopt leave-one-out experiment since this particular form of cross validation is an unbiased estimator of the generalization performance of classifier. It makes the best use of the available data and involves no random subsampling. Every time we pick out one sample (*l*_1 _= 1 or *l*_2 _= 1) from the training data and try to classify it correctly. And by doing *m*_1 _+ *m*_2 _times test we add *m*_1 _+ *m*_2 _constraints.

#### Linear programming approximation

In general, mixed integer linear programming is difficult to solve. To ensure the computational efficiency, mixed ILP in Equation (16) can be relaxed to the corresponding linear programming (LP). Linear programming is the simplest type of mathematical programming and has been widely used in systems biology study [5,13,14]. Therefore, sophisticated algorithm can be adopted to efficiently solve this LP. In terms of computational complexity, the proposed approach makes the computation of biomarker tractable. Finally we construct the LPFS model in Equation (17).

(17) $$\begin{array}{c} \underset{x,y}{\text{min}}\;\;\;\;\;\;\;\;\;\;\;\;\;\;\;\;\sum_{i=1}^{n}x_{i}+\lambda \sum_{i=1}^{l_{1}+l_{2}}y_{i}\\ s.t.\;\;\;\;\;\;\;\;\sum_{i=1}^{n}\left|p_{li}-\sum_{j=1}^{m_{1}-1}t_{ji}/\left(m_{1}-1\right)\right|x_{i}-y_{l}<\sum_{i=1}^{n}\left|p_{li}-\sum_{j=1}^{m_{2}}f_{ji}/m_{2}\right|x_{i}\\ \;\;p_{l}=\left(p_{l1},s_{l2},\cdots \;,p_{ln}\right)\in T,l\in \left\{1,2,\cdots \;,l_{1}\right\},t_{k}=\left(t_{k1},t_{k2},\cdots \;,t_{kn}\right)\in T\backslash \left\{p_{l}\right\},k\in \left\{1,2,\cdots \;,m_{1}\right\}\backslash \left\{l\right\}\\ \;\;\;\;\;\;\;\;\;\sum_{i=1}^{n}\left|p_{li}-\sum_{j=1}^{m_{1}}t_{ji}/m_{1}\right|x_{i}+y_{l}>\sum_{i=1}^{n}\left|p_{li}-\sum_{j=1}^{m_{2}-1}f_{ji}/\left(m_{2}-1\right)\right|x_{i}\\ \;\;p_{l}=\left(p_{l1},s_{l2},\cdots \;,p_{ln}\right)\in F,l\in \left\{1,2,\cdots \;,l_{2}\right\},f_{k}=\left(f_{k1},f_{k2},\cdots \;,f_{kn}\right)\in F\backslash \left\{p_{l}\right\},k\in \left\{1,2,\cdots \;,m_{2}\right\}\backslash \left\{l\right\}\\ \;\;\;\;\;\;\;\;\;\;\;x_{i}\geq 0,\;i\in \left\{1,2,\cdots \;,n\right\},y_{j}\geq 0,\;j\in \left\{1,2,\cdots \;,l_{1}+l_{2}\right\} \end{array}$$

After relaxing to continuous value, the value of the optimal solution of *x*_*i *_(LPFS score) indicates the importance of feature *i *in the nearest centroid classifier. It should be noted that we can use other distance definitions instead of *L*_1 _in our model to achieve the nonlinear classification effect. The parameter λ can be determined by checking the output leave-one-out predictive accuracy. We also notice that LPFS model can be extended to multi-classification task and n-fold cross validation.

### Metabonomics profiling by ^1^H NMR spectra

Venous blood (3ml) was collected into a heparin sodium tube and the plasma was collected by centrifugation at 1000× g at 4°C for 10 minutes. An aliquot of 300 *μ*l plasma was mixed with 250 *μ*l D_2_O and 50 *μ*l TSP (3-trimethylsilyl-^2^H_4_-propionic acid) in D_2_O (1 mg/ml) in 5 mm NMR tube. The D_2_O provided a field-frequency lock solvent for the NMR spectrometer and the TSP served as an internal reference of chemical shift. ^1^H NMR spectra of the plasma samples were acquired on a Varian INOVA 600 MHz NMR spectrometer at 27°C by using Carr-Purcell-Meiboom-Gill (CPMG) spin-echo pulse sequence. with a total spin-spin relaxation delay (2n*τ*) of 320 ms. The free induction decays (FIDs) were collected into 32K data points with a spectral width of 8000 Hz and 64 scans. The FIDs were zero-filled to double size and multiplied by an exponential line-broadening factor of 0.5 Hz prior to Fourier transformation (FT). In addition, diffusion-edited experiments were also carried out with BPP-LED (bipolar pulse pair longitudinal eddy current delay) pulse sequence [15,16]. The gradient amplitude was set at 35.0 G cm^-1^, with a diffusion delay of 100 ms. A total of 128 transients and 16k data points were collected with a spectral width of 8000 Hz. A line-broadening factor of 1 Hz was applied to FIDs before Fourier transformation.

All plasma ^1^H NMR spectra were manually phased and baseline corrected using VNMR 6.1C software (Varian, Inc.). For CPMG spectra, each spectrum over the range of 0.4 to 4.4 was data-reduced into integrated regions of equal width (0.01 ppm). For BPP-LED data, each spectrum over the range of 0.1 to 6.0 was segmented into regions of equal width (0.01 ppm). The regions containing the resonance from residual water (4.6-5.1) were excluded. The integral values of each spectrum was normalized to constant sum of all integrals in a spectrum in order to reduce any significant concentration differences between samples [17,18]. Identification of metabolites in spectra was accomplished based on literatures and the Chenomx NMR Suite 5.0 (Chenomx, Calgary, Canada).

## Results

### Metabonomics data generation

To investigate the acupuncture treatment effects, we originally obtained metabonomics data of plasma metabolites in healthy males at five meridian points using Proton NMR. Proton NMR (also named as Hydrogen-1 NMR, or ^1^H NMR) applies nuclear magnetic resonance in NMR spectroscopy with respect to hydrogen-1 nuclei within the molecules of a substance, in order to determine the structure of the molecules [19].

As a result, most organic compounds are characterized by chemical shift values, which are usually expressed in parts per million (ppm) by frequency and are in the range +14 to -4 ppm. Chemical shift values are not precise, but typically they are regarded mainly as orientational. The exact value of chemical shift depends on molecular structure and the solvent in which the spectrum is being recorded. These chemical shift values can be mapped to eight metabolic subsets (amino acids, carbohydrates, energy, glycans, lipids, nucleotides, secondary metabolites/xenobiotics, vitamins, and cofactors). In our experiment, 400 chemical shift values are measured for their concentration in plasma, and mathematically every sample is represented by a vector in 400 dimensional space.

**Figure 3:**
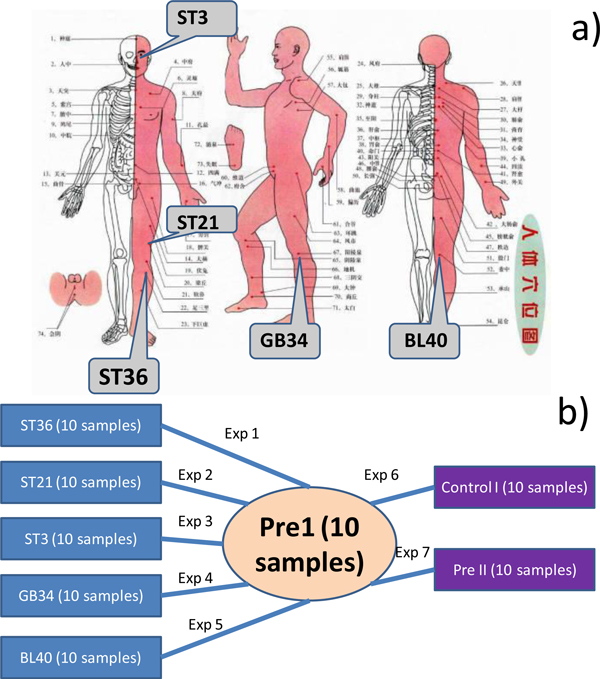
**Overall design of the biomarker identification experiments**. a) Metabolite profiles are originally generated by ^1^H NMR from five acupuncture points (Zusanli (ST36), Liangmen (ST21), Juliao (ST3), Yanglingquan (GB34), and Weizhong (BL40)). b) The metabolite profiles are grouped into 7 sets and the biomarker identification problem is designed as 7 binary classification experiments.

Fifty healthy young males were randomly allocated to Zusanli (ST36), Liangmen (ST21), Juliao (ST3), Yanglingquan (GB34), and Weizhong (BL40) groups (The locations of the meridian points are shown in Figure 3a. Among the five points, Zusanli, Liangmen, and Juliao are on the same meridian.). Each group contains 10 persons. Inside each group the corresponding meridian points were separately acupunctured for 5 consecutive days. In addition, twenty healthy young males are recruited as the blank control groups. All the twenty people are measured before the start of 5 consecutive days and additionally ten of them are measured after 5 consecutive days. Fasting venous blood was taken in all the subjects. Plasma metabolites were measured by ^1^H NMR to derive metabolic profiles (see details in Method Section). Furthermore to exclude possible noises, all the seventy males are strictly trained to make sure their metabolic profiles are measured in very similar conditions. The detailed experimental method can be found in [20]. In summary, we have 80 samples grouped into Zusanli (10 samples, acupuncture point ST36), Liangmen (10 samples, acupuncture point ST21), Juliao (10 samples, acupuncture point ST3), Yanglinquan(10 samples, acupuncture point GB34), Weizhong (10 samples, acupuncture point BL40), Control I (10 samples, normal people measured after the consecutive 5 days), and Control II (20 samples, normal people measured before the consecutive 5 days).

### Classification experiments design

With the data, we design experiments to identify biomarkers for the acupuncture treatment of each meridian point. The overall design of biomarker identification experiments is shown in Figure 3b. We categorize eighty samples into 8 groups shown as the circles in Figure 3b. ST36, ST21, ST3, GB34, and BL40 each has 10 samples. The 20 samples in Control II are naturally decomposed into two groups with equal size, Pre1 (10 samples with follow-up measurement after 5 days) and Pre2 (10 samples without follow-up measurement). Treating the Pre1 as the common control set, we have seven classification tasks (Exp1 to Exp7) shown as the lines in Figure 3b. For example, task Exp1 tries to identify a subset of metabolites to classify Pre1 as the control and ST36 as the case. In this way, Exp1 to Exp5 aim to identify the biomarkers for acupuncture treatment on ST36, ST21, ST3, GB34, and BL40 respectively. While Exp6 tries to capture the metabolite change by 5 consecutive days without acupuncture. And Exp7 tries to test if there are significant metabolite change for the people under similar condition. Exp6 and Exp7 serve as the control studies to guarantee the significance of our result.

### Global characterization of the data

**Figure 4:**
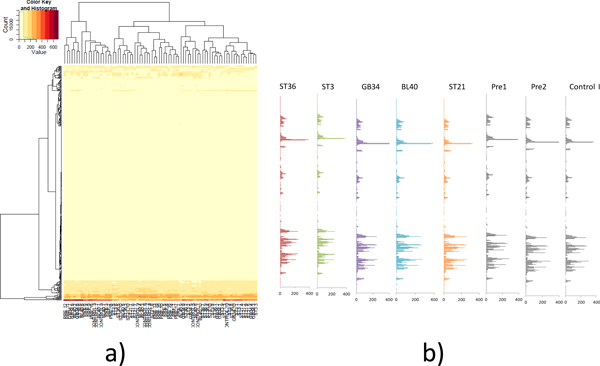
**Global characterization of the metabolite profiles**. a) Hierarchical clustering of the metabolic profiles of the 80 samples. b) Centroids for the seven datasets. The horizontal units are expression values for the metabolites. The metabolites are sorted by their chemical shift values.

We first perform hierarchical clustering on the 80 metabolic profiles. The results are shown in Figure 4a. If the samples can be clearly discriminated by global pattern, the 80 samples should be clustered by with or without acupuncture treatment and then by their meridian points. However, all the sample labels are mixed in the clustering result (Figure 4a) and we cannot see clearly boundaries.

Furthermore, we calculate the centroids for the seven groups of samples in Figure 4b by averaging the 10 samples for their metabolite expression values. These centroids are plotted side by side in Figure 4b, which shows that these centroids are very similar and it's very difficult to detect the global difference.

The above results together demonstrate that global pattern in metabolic profiles cannot discriminate the Zusanli, Yanglingquan, Liangmen, Juliao, Weizhong, Pre1, Pre2, and Control I groups. Thus it is necessary to find the local pattern in the profile data. Our strategy is to find a subset of metabolites as biomarkers to achieve clear discrimination.

### Comparison with other approaches

Before we conduct the acupuncture biomarker identifications, we benchmark our LPFS method by comparing with several existing state-of-the-art methods. There are many existing feature selection methods and they can be roughly categorized into three types, filter, wrapper, and embedded methods. To make the comparison simple and comprehensive, we pick out some representative methods in each type to compare in the same dataset.

Filter methods select features as a preprocessing step and feature selection part is independent of a machine learning algorithm (classifier). This is computationally efficient. Fold change and t-test are the simplest and popular methods to identify biomarker. They are usually the representative methods for filter methods.

Let *x*_*ij *_and *y*_*ij *_denote the log expression values of metabolite *i *in sample *j *in the case and control, respectively. We define the ordinary two-sample t-statistic [21] as

(18) $$T_{i}\frac{{\bar{x}}_{i}-{ȳ}_{i}}{s_{i}}$$

Where ${\bar{x}}_{i},{ȳ}_{i}$, and *s*_*i *_are the mean of case, mean of control, and the standard deviation of the samples for metabolite *i*. From t-statistic *T*_*i *_we can easily calculate a p-value. Usually a feature is selected if its corresponding p-value is smaller than a predefined threshold 0.05.

The standard definition of the fold-change [21] for metabolite *i *is

(19) $$FC_{i}=\frac{{\bar{{}^{{}^{\prime }x}}}_{i}}{{\bar{{}^{{}^{\prime }y}}}_{i}}$$

Where ${\overset{´}{x}}_{ij}$ and *ý*_*ij *_are the raw expression values of metabolite *i *in sample *j *in the case and control, respectively. In our implementation, we computes the difference of means (i.e. the fold-change for log-transformed data) and then rank the metabolites by their absolute values. We choose a cutoff 2 to select the significant ones.

On the other hand, wrapper method ranks features based on their effects on classification accuracy. It takes dependencies of the feature subset on the learning algorithm into account and is computationally more demanding. Support Vector Machine-Recursive Feature Elimination (SVM-RFE) is one of the most successful wrapper method based algorithm in the feature selection [22]. SVM-RFE conducts feature selection in a sequential backward elimination manner, which starts with all the features and discards one feature at a time by checking the SVM accuracy. It has been widely used and extended in high-dimensional data analysis [23]. We compare our LPFS method with SVM-RFE in metabolite data.

Since our LPFS method is an embedded method and simultaneously optimize classification accuracy and the number of selected features, we specifically choose to compare with an existing method with similar strategy, called sparse multinomial logistic regression approach (SMLR). It was developed to jointly and simultaneously identify the optimal nonlinear classifier, and select the optimal set of features via the optimization of a single posterior objective function (see [24] and [25]). SMLR has been extensively applied in problems in systems biology [26]. SMLR is freely available at http://www.cs.duke.edu/~amink/software/smlr/ and we take the default values for the parameters in our calculation.

Without loss of generality, we take Exp1 in Figure 3b as an example to identify a subset of metabolites to discriminate ST36 and Pre1. We select biomarkers from the metabolic profiles and compare the results in two ways.

**Figure 5:**
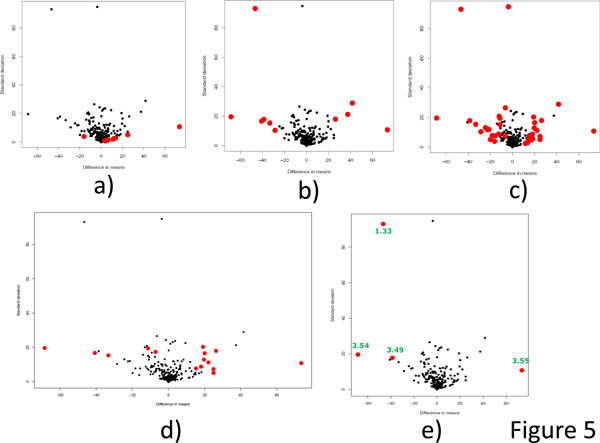
**Methods comparison via volcano like plots**. Comparison of our LPFS method with existing methods regarding to the identified biomarkers. All the 400 metabolites are plotted into a two dimensional plane. The selected biomarkers are highlighted in red. The x-axis denotes the difference of means and the y-axis denotes the standard derivation. Good biomakers should locate either in the left bottom corner or in the right bottom corner. a) volcano like plot of t-test method. The top 10 features are in red. b) volcano like plot of fold change method. The top 10 features are in red. c) volcano like plot of SMLR method. d) volcano like plot of SVM-RFE method. e) volcano like plot of our LPFS method.

Firstly, we compare different methods in Figure 5, by assessing the quality of the selected biomarkers. We simply plot all the metabolites by their standard derivation versus the difference of mean expression value. This is a scatter-plot used in [27], which plots significance versus fold-change on the y- and x-axes, respectively. Conceptually, it is very similar to the volcano plot in statistics [28]. Plotting points in this way results in two regions of interest in the plot: those points that are found towards the bottom of the plot and far to either the left- or the right-hand side. These represent values that display large magnitude fold changes as well as high statistical significance with small standard derivation.

The t-test based method identifies 172 metabolites if we choose a cutoff 1.73 (corresponding p-value 0.05). A strict threshold will still select 84 metabolites with cutoff 2.84 (corresponding p-value 0.005). We list the top 10 in Table 1 and plot them in Figure 5a. We find that the ordinary t-statistic selects metabolites with low standard deviations.

**Table 1:** The top ten identified biomarkers by different methods on the ST36 meridian point.

Student t-test			Fold change			SMLR		SVM-RFE		Our LPFS method			
**ID**	**ppm**	**t-score**	**ID**	**ppm**	**FC-score**	**ID**	**ppm**	**ID**	**ppm**	**ID**	**ppm**	**LPFS score**	**Metabolite name**

86	3.55	15.29	86	3.55	73.48	45	4	86	3.55	86	3.55	0.015	
195	2.46	11.91	87	3.54	68.52	50	3.95	68	3.73	92	3.49	0.008	
251	1.9	11.07	308	1.33	46.58	52	3.93	87	3.54	87	3.54	0.006	a-glucose/glycine
45	3.96	10.96	310	1.31	41.61	58	3.87	69	3.72	308	1.33	0.002	lactate
229	2.12	10.86	70	3.71	40.75	60	3.85	102	3.39				
81	3.6	10.80	92	3.49	38.61	67	3.78	70	3.71				
18	4.23	10.03	293	1.48	37.45	68	3.77	295	1.46				
127	3.14	9.75	295	1.46	33.26	69	3.76	116	3.25				
17	4.24	9.71	71	3.7	28.55	70	3.75	229	2.12				
232	2.09	9.35	69	3.72	26.30	71	3.74	88	3.53				

The fold change based method identifies 159 metabolites if we choose a commonly used cutoff 2 [28]. And there are still 97 metabolites by choosing a cutoff 4. Again the top 10 are listed in Table 2 and plotted in Figure 5b. It's clear that the fold-changes select metabolites with large shifts between control and treatment.

**Table 2:** Identified biomarkers from different meridian points by our LPFS method.

Zusanli ST36			Liangmen ST21			Juliao ST3			Yanglingquan GB34			Weizhong BL40		
**Metabolite**	**ppm**	**ID**	**Metabolite**	**ppm**	**ID**	**Metabolite**	**ppm**	**ID**	**Metabolite**	**ppm**	**ID**	**Metabolite**	**ppm**	**ID**

	**3.55**	86		**2.11**	230		**3.55**	86		**3.55**	86		**3.78**	63
a-glucose/glycine	**3.54**	87		**0.88**	353	a-glucose/glycine	**3.54**	87	a-glucose/glycine	**3.54**	87		**3.99**	42
	**3.49**	92	histidine/taurine	**3.25**	116				threonine	**1.32**	309		**3.88**	53
lactate	**1.33**	308		**3.55**	86							lipid	**1.3**	311
			lactate	**1.33**	308							lysine/arginine	**1.91**	250
			a-glucose/glycine	**3.54**	87								**3.92**	49
				**3.2**	121								**3.49**	92
													**3.2**	121

While SMLR selects 37 features to achieve the 100% leave-one-out predictive accuracy. These 37 metabolites are plotted in Figure 5c and 10 of them (ranked by ID) are listed in Table 1. Figure 5c demonstrates that SMLR is quite efficient to cover almost all the metabolites with large fold change and small standard derivation. However, 37 metabolites is too much from the viewpoint of practical usage of biomarkers.

SVM-RFE selects 15 metabolites in total to achieve the 100% leave-one-out predictive accuracy. These metabolites are illustrated in Figure 5d and the top ten are listed in Table 1. Figure 5d shows that SVM-RFE favors the metabolites with small standard derivation.

Our LPFS method finally selects 4 features as the biomarkers to discriminate ST36 and Pre1. By using only 4 features we can achieve 100% leave-one-out predictive accuracy. To show these four important biomarkers are not dependent on the nearest centroid classifier, we use SVM to do five-fold cross validation, the predictive accuracy is still 100%. This demonstrates that we can select a small set of important features really matters by applying strong regularization. The selected 4 metabolites are listed in Table 1 and scatter plotted in Figure 5e. Figure 5e also shows that our LPFS method tends to reveal the metabolites with small standard deviation and large shifts, which exactly serves our requirement for good biomarker. The four metabolites are with ppm 3.55, 3.54, 3.49, and 1.33. ppm 3.54 and 1.33 are annotated as a-glucose/glycine and lactate respectively. The expression level of ppm 3.54, 3.49, and 1.33 goes down after acupuncture treatment while the expression level of ppm 3.55 goes up.

**Figure 6:**
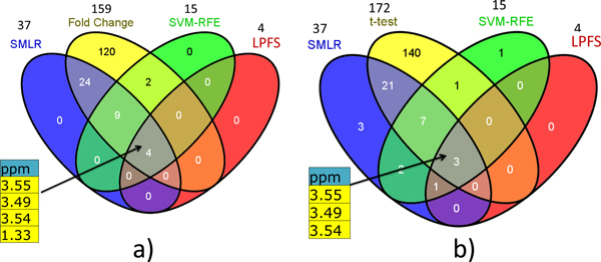
**Venn diagram for the results obtained by different methods**. a) Comparing t-test, SMLR, SVM-RFE, and LPFS methods by checking the overlaps of their selected biomarkers. b) Comparing fold change, SMLR, SVM-RFE, and LPFS methods.

Secondly, we compare the results of these five methods in a venn diagram in Figure 6. Figure 6a shows the overlap among the results from fold change, SVM-RFE, SMLR, and LPFS. The 4 metabolites selected by our LPFS, metabolites with ppm 3.55, 3.54, 3.49, and 1.33, also correctly selected by fold change, SVM-RFE, and SMLR. Interestingly, all the results obtained by SVM-RFE, SMLR, and LPFS are included in the results by fold change. Figure 6b shows the overlap among the results from t-test, SVM-RFE, SMLR, and LPFS. Again, metabolites with ppm 3.55, 3.54, 3.49 are consistently supported by SVM-RFE, SMLR, and t-test. Metabolite with ppm 1.33 is not included in the t-test result but supported by SVM-RFE and SMLR. SVM-RFE and SMLR also select some metabolites which are not included by t-test results. The venn diagram demonstrates that fold change method is more consistent with current feature selection methods when the sample number is not so large.

In addition to the overall venn diagram, the top ten biomarkers obtained by the t-test, fold change, SVM-RFE methods are summarized in Table 1. When comparing the overlap with top 10 list, LPFS still gets consistent results with other methods. Metabolite with ppm 3.55 has a t-test score 15.29 and fold change score 75.38. It is ranked the first by all the three methods with rank information. Metabolites with ppm 3.54 and 3.49 rank high in fold change, SVM-RFE. ppm 1.33 ranks high in the result obtained by fold change.

### Biological insights for the identified biomarkers

We then applied the proposed LPFS method to identify the biomarkers from the designed seven experiments. As a result, we identified 4, 7, 2, 3, and 8 biomarkers for the acupuncture treatment effects of ST36, ST21, ST3, GB34, and BL40 respectively. These selected biomarkers can achieve 100%,100%,100%,100%, and 95% leave-one-out cross validation accuracy. The results are summarized in Table 2. As expected, Exp7 fails to find any biomarkers. Exp6 finds several metabolites due to the fact that the expression values of these metabolites vary after consecutive 5 days. So we carefully check the obtained metabolites list by Exp6 and exclude these metabolites in our final results. Some biomarkers identified in Table 2 are annotated as glucose and lipid. Most of them need further investigation on their chemical structures and biological functions.

From Table 2, we can see that acupuncture at Yangming meridian points (including acupuncture points at ST36, ST21, and ST3) influence mainly plasma micromolecular metabolites and was closely related to energy metabolism pathway. Acupuncture at Yanglingquan influences mainly plasma macromolecular metabolites and is closely related to lipid metabolism and transport. Acupuncture at Weizhong doesn't largely influence plasma metabolites. This study suggests that Yangming meridian points have certain characteristics, which are different from those of both Yanglingquan and Weizhong. Metabonomics techniques based on ^1^H NMR and biomarker identification method provide experimental evidence for distinguishing between Yangming meridian points and other meridian points from the metabolic aspect. This fact may become a new useful information source to study the specificities of meridian points.

**Figure 7:**
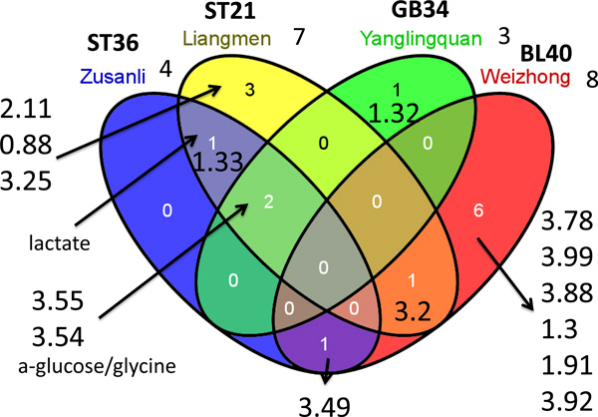
**Venn diagram for identified biomarkers from different acupuncture points**. The similarity and difference of those identified biomarkers from Zusanli, Liangmen, Yanglingquan, and Weizhong are shown in a venn diagram. The overlapped biomarkers are indicated by their ppm and known annotation.

To reveal the similarity and difference of the identified biomarkers regarding to meridian points, we calculate the overlaps of these biomarkers and present them in Figure 7. We found that Weizhong is slightly different from other meridian points. There is no overlapped metabolites for Weizhong and other meridian points. Compared to that, Zusanli, Yanglingquan, Juliao, and Liangmen are close to each other by sharing two common metabolites: ppm 3.54 and 3.55 (Juliao is not shown in Figure 7. The two biomarkers from Juliao are ppm 3.55 and 3.54 and totally included by Zusanli, Yanglingquan, and Liangmen). In them 3.54 is annotated as a-glucose/glycine. Among the five points, Zusanli, Liangmen, and Juliao are on the same meridian. From the venn diagram in Figure 7, we can clearly see this trend. Yanglingquan has a unique biomarker with ppm 1.32. Our biomarker analysis indicates that acupuncture has some common molecules in metabolic level. At the same time, acupuncture at different meridian points has different molecular response. Our result is consistent with the theory on specificities of meridian points.

**Figure 8:**
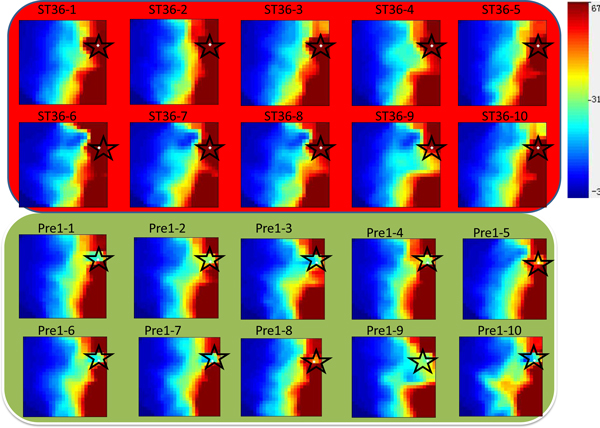
**Highlight the selected biomaker in 2D plot**. Metabolic sample is visualized as a two dimensional image. Each grid denotes a group of metabolites with similar profiles. Red color means the highly expressed metabolite group and blue color means the lowly expressed metabolite group. In particular, metabolite with chemical shift value 3.55 is highlighted in white color and indicated by the star.

Our results show that metabolite with chemical shift value 3.55 is clearly a common biomarker for ST36, ST21, ST3, and GB34. In Figure 8, we visualize the metabolic profiles as a two-dimensional graph and highlight this important molecule. The two dimensional graph, called the GEDI-"mosaics", provide a unique, one-glance visual engram that gives each high-dimensional sample a face. A characteristic of GEDI's analysis is that it does not prejudicate any particular structure in the data (such as clusters or hierarchical organization). Thus, it allows the researcher to use human pattern recognition to perform a global first-level analysis of the data [29] (GEDI is downloaded from http://www.childrenshospital.org/research/ingber/GEDI/gedihome.htm). It is clear that the highlighted metabolite has distinct expression value in case and control group (ST36 and Pre1 in Figure 8). This demonstrates the effectiveness of our biomaker identification method.

Importantly, our LPFS method reveals the metabolite with ppm 1.33 as a biomarker for meridian points ST36 and ST21. This molecule is annotated as lactate. Lactate has been extensively studied over years for many important functions. For example, the lactate has always been regarded as the central nervous system metabolic waste and a sign of hypoxia [30]. Also in recent years, evidences show that the role of lactate in brain energy metabolism should be re-recognized. Firstly, investigations find that lactate is not only a very important energy resource for brain, but a sensor of brain energy homeostasis [31]; secondly, lactate in adult brain mainly comes from astrocytes, and it is a collaborative carrier for neuron and astrocytes [32,33]; thirdly, lactate plays an important role in coupling energy metabolism and functional activity [34]; and lastly, lactate has neuroprotective effect in a number of pathological conditions. It's interesting to see lactate as a biomaker closely relating to acupuncture in our result. Whether lactate plays a substantial role in acupuncture effect at ST36 and ST21 needs follow-up biological experiments.

## Discussions and conclusions

Biomaker identification or feature selection considers the problem of constructing a prediction rule from only a feature-subset and accurately classifying the context of diagnosis and treatment observations (e.g. with vs. without acupuncture treatment). Such problems have become increasing important and quite general in genomics (identifying differentially expressed genes in microarray data), proteomics (finding promising protein marker from the mass spectrometry data), metabolics (selecting metabolite markers from NMR, GC-MS data), and other areas of computational biology. Due to the number of features is much larger than the number of observations, simple, highly regularized approaches are in pressing need. Here, we proposed a novel linear programming based feature selection (LPFS) model to address this important problem. The feature selection problem is cast into an optimization problem with two objectives, one is to minimize the number of chosen features and the other is to maximize the predictive accuracy. Mathematically the feature selection problem is formulated as a mixed integer linear programming problem. Then the model is further relaxed to linear programming to ensure the efficient identification of a feature-subset. We can solve the in-essence combinatorial optimization problem in a computational reasonable way. In summary, our LPFS method can select feature and learn the classifier in a joint way and we can select a small set of features by applying strong regularization. Our methodology is general and can be easily applied to other scenarios [35].

We extensively compared our LPFS method with existing methods in the real datasets on acupuncture treatment at different acupoints. We find that, 1). Our method can select the fewest features while achieve accurate predictions. 2). Our method is free of arbitrary threshold choice. 3). Close check of the selected feature shows that our method can identify those biological meaningful features. 4). In addition, the cross-validation results show that our method can achieve relatively high accuracy in prediction.

Prior information allows further improvement of our method. Currently the identified biomarkers are independent to each other. We can move further step to interpretation by considering a group of biologically meaningful biomarkers. For example, we can incorporate the network information (interactions among features) into the feature selection procedure. As a result, a pathway or modules in the network will be finally selected instead of single molecule as the biomarker, so called network biomarker. We note that prior information can be easily incorporated into our optimization model either by adding some constraints or penalizing in the objective function.

In this paper, the biomarker identification for each acupuncture point is treated as a single binary classification task. We then compare the revealed biomarkers for their similarity and difference across different acupuncture points. We note that a multi-classifier can be developed to systematically integrate all the profiles from different points together. This topic is in progress as our further direction.

Finally, the metabolic profile is known for its high variance. We note that the main source of variance is from NMR technology instead of acupuncture effect [36]. To maximally reduce the variance from metabolic profile, we carefully design our experiments. Firstly, we used the relatively stable blood samples instead of urine sample. Secondly, we specifically use the Pareto scaling and orthogonal signal correction (OSC) method [37-39] to normalize the raw data, which will reduce the variance of samples inside each group and enhance the differences among groups. Even with all these efforts, the remaining high variance may due to the change of environments and conditions and will eventually prevent the high accuracy for identification of biological meaningful biomarkers. In addition to variance, the limited number of sample in our study may also bring some potential effects on the results. From this viewpoint, these identified biomarkers should be carefully validated for their biological functions. Also, additional control study should be carefully designed to exclude other possible cofactors. Further integration of the data from other levels, such as gene expression and proteomics levels, will improve the robustness of the identified biomarker.

## Competing interests

The authors declare that they have no competing interests.

## Authors' contributions

YW proposed the computational method. QFW, XZY, SGY, and FRL designed the experimental study and generated the data. YW, CC, LYW, and XSZ implemented the method, performed the experiments and analyzed the data. YW and QFW wrote the manuscript. All authors revised the manuscript and approved the final version.
